# Expression profiling in canine osteosarcoma: identification of biomarkers and pathways associated with outcome

**DOI:** 10.1186/1471-2407-10-506

**Published:** 2010-09-22

**Authors:** Liza E O'Donoghue, Andrey A Ptitsyn, Debra A Kamstock, Janet Siebert, Russell S Thomas, Dawn L Duval

**Affiliations:** 1 Department of Clinical Sciences, Colorado State University, Fort Collins, Colorado, USA; 2 Department of Microbiology, Immunology and Pathology, Colorado State University, Fort Collins, Colorado, USA; 3 CytoAnalytics, Analytical Services, Denver, Colorado, USA; 4 The Hamner Institutes for Health Sciences, Research Triangle Park, North Carolina, USA

## Abstract

**Background:**

Osteosarcoma (OSA) spontaneously arises in the appendicular skeleton of large breed dogs and shares many physiological and molecular biological characteristics with human OSA. The standard treatment for OSA in both species is amputation or limb-sparing surgery, followed by chemotherapy. Unfortunately, OSA is an aggressive cancer with a high metastatic rate. Characterization of OSA with regard to its metastatic potential and chemotherapeutic resistance will improve both prognostic capabilities and treatment modalities.

**Methods:**

We analyzed archived primary OSA tissue from dogs treated with limb amputation followed by doxorubicin or platinum-based drug chemotherapy. Samples were selected from two groups: dogs with disease free intervals (DFI) of less than 100 days (n = 8) and greater than 300 days (n = 7). Gene expression was assessed with Affymetrix Canine 2.0 microarrays and analyzed with a two-tailed t-test. A subset of genes was confirmed using qRT-PCR and used in classification analysis to predict prognosis. Systems-based gene ontology analysis was conducted on genes selected using a standard J5 metric. The genes identified using this approach were converted to their human homologues and assigned to functional pathways using the GeneGo MetaCore platform.

**Results:**

Potential biomarkers were identified using gene expression microarray analysis and 11 differentially expressed (p < 0.05) genes were validated with qRT-PCR (n = 10/group). Statistical classification models using the qRT-PCR profiles predicted patient outcomes with 100% accuracy in the training set and up to 90% accuracy upon stratified cross validation. Pathway analysis revealed alterations in pathways associated with oxidative phosphorylation, hedgehog and parathyroid hormone signaling, cAMP/Protein Kinase A (PKA) signaling, immune responses, cytoskeletal remodeling and focal adhesion.

**Conclusions:**

This profiling study has identified potential new biomarkers to predict patient outcome in OSA and new pathways that may be targeted for therapeutic intervention.

## Background

Osteosarcoma (OSA) is the most common malignant primary bone tumor of children and accounts for roughly 5% of all childhood cancers in the United States. Characteristically, OSA is found in the metaphyseal regions of long bones in the appendicular skeleton. More than 15% of patients present with clinically detectable pulmonary metastases and it is estimated that 80% or more have micrometastases at presentation [1]. Advances in treatment such as multi-agent chemotherapy have improved prognosis over the last several decades with five-year survival rates up to 70%. Despite these advances, patients that present with metastases or those whose tumors are refractory to neoadjuvant chemotherapy continue to have a poor prognosis [1]. This suggests that within the same histologic type of tumor, different genetic mechanisms may be operating, altering response to chemotherapy and metastatic capability in some tumors.

Osteosarcoma is also the most common primary bone malignancy in dogs. The majority of these tumors occur in the appendicular skeleton of middle-aged large and giant breeds. Roughly 10,000 new cases of OSA are identified in dogs annually. Standard treatment involves amputation or limb-sparing surgery followed by adjuvant chemotherapy with doxorubicin, a platinum-based drug, or a combination of the two drug types [2]. Median disease-free interval (DFI) following amputation has ranged from 165 to 470 days depending on adjuvant chemotherapy protocol and study size [3-7]. Median survival time in dogs undergoing amputation alone ranges from 134 to 175 days [3-7]. Like their human companions, pulmonary metastases are typically the cause of terminal morbidity. It has been suggested that up to 90% of canine patients may present with microscopic metastases that are undetectable via routine imaging [2]. The high variability in DFI suggests that canine OSA exhibits variable metastatic capability, rate and/or resistance to adjuvant chemotherapy, similar to the disease in humans.

Canine OSA shares many features with human OSA, making dogs a valuable comparative model. Pet dogs develop OSA primarily in the metaphyseal regions of long bones, as do human patients. The lesions are histologically identical [2]. The similarities between the molecular characteristics of human and canine OSA have been established (see [8] for review). Furthermore, Thomas and colleagues recently demonstrated that some of the same genetic aberrations identified in human OSA are also seen in canine OSA with both breed-dependent and independent associations [9]. Among the genetic changes identified, Wilms tumor 1 (*WT1*), tumor protein p53 *(TP53)*, cyclin-dependent kinase inhibitor 2A *(CDKN2A)*, phosphatase and tensin homolog *(PTEN) *and retinoblastoma 1 *(RB1) *tumor suppressors as well as v-myc myelocytomatosis viral oncogene homolog (*MYC) *and v-kit Hardy-Zuckerman 4 feline sarcoma viral oncogene homolog *(KIT) *oncogenes were shown to be affected by cytogenetic abnormalities in 76% of their samples [9]. Similarly, comparative analysis of gene expression profiles in human and canine OSA determined that the diseases were indistinguishable by hierarchical clustering [10]. Treatment and chemotherapeutic regimens are also similar with the notable exception that most amputee dogs do not undergo neoadjuvant chemotherapy, so tumors collected at the time of amputation are naïve to drugs. Dogs also provide a valuable model system in that their tumors arise "naturally," they share an environment with humans, and they metabolize drugs at a similar rate. Finally, dogs are more genetically diverse than mouse model systems and share more genetic homology with humans than mice [8]. Thus, genetic prognostic screening in dogs has strong potential applicability to the human disease [11].

In recent years, it has become clear that the tumor microenvironment plays a strong role in metastatic events even if metastatic subclones are only a small proportion of tumor cells [12,13]. For example, van de Vijver and colleagues demonstrated that gene expression analysis of primary tumors can divide breast cancer patients into "good" and "poor" prognostic groups based on the tumors' intrinsic metastatic ability [14]. Thus, gene expression profiles of primary tumors provide information about metastatic potential and patient prognosis even if distant disease is not detectable or present.

Gene expression analysis of primary tumors can also elucidate novel chemotherapeutic targets by defining individual gene changes and/or whole pathway derangements [15,16]. Identification of such differences between "good" and "poor" prognostic groups in OSA will allow for more personalized treatment of disease based on an individual's tumor expression profile.

The current study utilized Affymetrix GeneChip Canine Genome 2.0 arrays to explore differences in gene expression between primary OSA tumors taken from client dogs with a DFI of less than 100 days ("poor responders") and those with a DFI greater than 300 days ("good responders") following definitive treatment and chemotherapy. Individual genes with significant changes in expression were validated using qRT-PCR and explored for their ability to correctly classify good and poor responders using four different machine learning schemes. A broader, systems approach was used to identify changes in groups of interacting genes or pathways that may contribute to metastatic progression and resistance to therapy. We have found evidence of altered expression of several genes and pathways and have verified that the Hedgehog signaling pathway is comparatively downregulated in the poor responding group.

## Methods

Chemotherapy-naïve primary tumor samples were selected from the Colorado State University Animal Cancer Center's tissue archive based on the criteria that the patient had undergone surgical amputation followed by chemotherapy with doxorubicin and/or a platinum-based drug (Table 1). Limb-spare surgical samples were excluded from the study as differences in DFI are associated with post operative infections common to the procedure [17,18]. Samples were collected at the time of amputation with the written consent of the owners (between 1996 and 2006), flash-frozen in liquid nitrogen and stored at -80°C. Disease-free intervals (DFI) were calculated based upon the presence of metastatic disease and samples were divided into cohorts of DFI < 100 days and DFI > 300 days. These cohorts were defined to select samples distant from the median DFI of 200 days so that expression differences could be analyzed in very good and very poor responders.

**Table 1:** Study Population.

Unique ID	DFI	Age at Dx (yrs)	Sex	Breed	Tumor Site	Tumor Subtype	Chemotherapy Received
184844	40	4.4	MC	Greyhound	L Prox Humerus	Osteoblastic	Doxorubicin
208911	60	8.0	FS	Doberman	L Prox Humerus	Giant cell	Carboplatin
173175	69	5.0	MC	Rottweiler	L Dist Femur	Osteoblastic	Cisplatin
223986	77	7.0	MC	Greyhound	L Dist Femur	Osteoblastic	Carboplatin
153599	90	9.0	FS	Mix	L Tibia	Giant cell	Cisplatin
222189	91	6.1	FS	Greyhound	L Prox Humerus	Osteoblastic	Doxo & Carbo
204714	94	8.0	FS	Greyhound	L Prox Tibia	Giant Cell	Doxorubicin
208756	95	10.2	FS	Labrador Ret.	L Dist Humerus	Osteoblastic	Cisplatin
146719	97	8.8	MC	Mix	R Dist Femur	Fibroblastic	Doxorubicin
212759	97	10.8	MC	Golden Ret.	L Prox Humerus	Osteoblastic	Doxorubicin
177466	307	7.6	FS	Mix	L Dist Radius	Osteoblastic	Cisplatin
188084	329	10.4	MC	Rottweiler	R Dist Radius	PD	Doxorubicin
190030	356	13.4	MC	Mix	L Dist Humerus	Osteoblastic	Doxorubicin
180223	384	11.5	FS	Mix	R Prox Femur	Osteoblastic	Cisplatin
208513	467	7.1	MC	Greyhound	L Prox Humerus	Osteoblastic	Doxorubicin
180119	619	10.4	FS	Mix	R Dist Femur	Osteoblastic	Cisplatin
193231	694	12.4	MC	Mix	L Dist Radius	Osteoblastic	Doxorubicin
174513	734	10.1	FS	Malamute	L Dist Radius	Osteoblastic	Doxo & Carbo
155214	787	8.7	MC	Labrador Ret.	R Tibia	Osteoblastic	Doxorubicin
168327	885	8.0	FS	Golden Ret.	L Dist Radius	Osteoblastic	Carboplatin

DFI = disease-free interval, Dx = diagnosis, MC = castrated male, FS = spayed female, L = left, Dist = distal, Prox = proximal, R = right, PD = poorly-differentiated, "Doxo & Carbo" = Doxorubicin and Carboplatin combination therapy,

Samples were freeze-fractured, homogenized, extracted with Trizol reagent (Invitrogen, Carlsbad, CA, USA) and purified with RNeasy clean up (Qiagen, Valencia, CA, USA) per the manufacturers' protocols. Resultant RNA was quantified via spectrophotometry and assayed for quality on Agilent (Santa Clara, CA, USA) and Bio-Rad (Hercules, CA, USA) bioanalyzers at the Rocky Mountain Regional Center for Excellence (RMRCE) Genomics Core at CSU. Only samples exhibiting minimal degradation as evidenced by RNA Integrity Numbers (RIN) greater than 8 were used for microarrays.

Eight samples from each DFI cohort were selected and array analysis with GeneChip Canine 2.0 Genome Arrays (Affymetrix, Santa Clara, CA, USA) was performed in two batches (batch 1, n = 6; batch 2, n = 10) at CSU's RMRCE Genomics Core per Affymetrix protocols. One sample was removed from analysis after data collection based upon pathologist review and review of hospital records that determined the sample was not OSA but hyperreactive osteoid tissue. Briefly, the One-Cycle Target Labeling and Control Reagents package (Affymetrix, Santa Clara, CA, USA) was used to synthesize cDNA from total RNA spiked with prokaryotic Poly-A RNA as a control. The Sample Cleanup Module (Affymetrix, Santa Clara, CA, USA) was used to purify the cDNA which was then used for synthesis of biotin-labeled cRNA. cRNA was purified, quantified and fragmented before hybridization with the GeneChips. Hybridized chips were washed, stained using streptavidin-conjugated phycoerythrin dye (Invitrogen, Carlsbad, CA, USA) and enhanced with biotinylated goat anti-streptavidin antibody (Vector Laboratories, Burlingame, CA, USA) using an Affymetrix GeneChip Fluidics Station 450 and Genechip Operating Software. The Affymetrix GeneChip scanner 3000 was used to acquire images.

Microarray data was preprocessed using Probe Logarithmic Intensity Error (PLIER) estimation [19] and Robust Multichip Average (RMA) [20] algorithms with log_2 _transformations. PLIER and RMA methods were compared as part of the data discovery. A standard unpaired 2-tailed t-test with a false discovery rate correction for multiple comparisons was used. Uncorrected p-values were used to rank probesets. CIMminer was used to generate clustered images of the data with the following parameters: unsupervised clustering on both axes, average linkage and Euclidean distance [21]. Microarray data has been made available through NCBI's Gene Expression Omnibus (GEO) and can be accessed via accession number GSE24251.

Quantitative RT-PCR was performed on an expanded set of 20 OSA samples including the same 15 samples used in the array analysis plus an additional five samples that met the selection criteria of amputation, chemotherapy, appendicular location of tumor and DFI (n = 3 in the DFI > 300 cohort and n = 2 in the DFI < 100 cohort). These additional 5 samples increased the number of samples in each cohort to ten. The sample set was expanded so that expression of genes of interest could be assessed in independent samples in addition to those from the microarray study. cDNA was synthesized using the QuantiTect Reverse Transcription Kit (Qiagen, Valencia, CA, USA) with 1 μg input RNA. Quantitative real time PCR was performed in duplicate using iQ SYBR Green Supermix (Bio-Rad, Hercules, CA, USA) and 25 ng equivalent RNA input in 25 μL reactions on a Stratagene Mx3000P instrument. Primers (Table 2) were designed based upon NCBI RefSeq mRNA sequences with PrimerQuest (Integrated DNA Technologies, Coralville, IA, USA) and were cross-checked for specificity using UCSC In-Silico PCR [22,23]. Where possible, primers were designed to be intron spanning and in a central region of the gene. Primers were designed to amplify all possible isoforms noted in NCBI and were not specific to the Affymetrix probe region. Expression levels were normalized to hypoxanthine phosphoribosyltransferase 1 (HPRT1) expression as it was consistently expressed at a moderate level in our arrays and has previously been used as a canine housekeeping gene [24] (primer sequences courtesy of Dr. Luke Wittenburg, CSU). Standard curves, dissociation curves and amplification data were collected using Mx3000P (Stratagene, La Jolla, CA, USA) software and analyzed with the 2^(-ΔΔCt) ^method [25] followed by an unpaired 2-tailed t-test as well as REST2009 software [26,27]. In all cases, amplification efficiencies were greater than 90%. Quantitative RT-PCR products were electrophoresed on a 2% agarose gel in 1× TBE and visualized under UV light with ethidium bromide to verify product size.

**Table 2:** Primer sequences and amplicon sizes for selected genes.

Primer	Sequence (5' to 3')	Size of Amplicon
HPRT1 S	TGC TCG AGA TGT GAT GAA GG	191 bp
HPRT1 AS	TCC CCT GTT GAC TGG TCA TT	
		
ADHFE1 S	CCA ACA GTG GCT TCG ATG TGC TTT	104 bp
ADHFE1 AS	TGC TGG CCG AGT GAT AGG ATT TGA	
		
AGTR1 S	TGA CTT TGC CAC TAT GGG CTG TCT	178 bp
AGTR1 AS	AGG CGG GAC TTC ATT GGA TGA ACA	
		
CCDC3 S	TGA ACC AGA AGC TCA GCG AGA AGT	162 bp
CCDC3 AS	TAG ATT CCC TGG CAA GAG GCA ACA	
		
DHH S	ACA ACC CGG ACA TCA TCT TCA AGG	109 bp
DHH AS	ATG TTC ATC ACC GCA ATG GCC AAG	
		
FBP1 S	TCC TGT ACC CAG CGA ACA AGA AGA	89 bp
FBP1 AS	TGC CTT CTC CAT GAT GTA GGC CAT	
		
IGF2 S	TCG TGG AAG AGT GCT GTT TCC GTA	154 bp
IGF2 AS	TCG TAT TGG AAG AAC TTG CCC ACG	
		
IMP1 S	TTG CAG AAT TTG ACA GCG GCT GAG	118 bp
IMP1 AS	TTT GGT GCA GCT GCT TAA CTT GGG	
		
NDRG2 S	ATA AGT CTT GCT TCC AGC CGC TCT	183 bp
NDRG2 AS	TCA GGT ACT GCA GAA TGC AAG GGA	
		
PTCH2 S	CAT ATT CCT GCT GGC ACA TGC CTT	229 bp
PTCH2 AS	GAA GAC AAG CAT CAC GGC TGC AAA	
		
SCN1B S	TCG TGG CAG AGA TGG TTT ACT GCT	121 bp
SCNIB AS	ACA CCC GTA CAG TTC TCC TTG CTT	
		
SMO S	TGG TGC TCA TTG TGG GAG GTT ACT	210 bp
SMO AS	ACT CAG CCT GGT TGA AGA AGT CGT	

S = sense, AS = antisense, bp = base pairs

The pathway analysis pipeline used in this study has been previously described [28]. Briefly, the University of Pittsburgh Gene Expression Data Analysis suite (caGEDA) [29] with a standard J5 metric, a threshold of 4 and a jackknife of 4 was used to select unique genes for pathway analysis following both PLIER and RMA preprocessing. The DAVID Gene ID conversion tool was used to link canine identifiers to their human counterparts [30,31] and identifiers absent from the database were hand-annotated by BLAST and BLAT comparisons of the target sequence; GeneGo MetaCore was used to assign functional pathways. Pathways were assigned independently to PLIER and RMA preprocessed data and the resulting pathways were compared.

WEKA software was used to generate classification models to test the analytical value of qRT-PCR-derived expression changes [32]. Classification models were built using a Support Vector Machine (SVM), a J48 decision tree, and logistic regression [33]. Models were generated with the full (n = 20) data set and tested for sensitivity and specificity using stratified tenfold cross-validation. Tenfold cross-validation randomly selects 90% of the data for training the model, and uses the other 10% of the data to test the model. The process is repeated ten times and the ten model error rates are averaged to compute an overall error rate.

## Results

### Tumor Donors

The DFI < 100 group was composed of 5 castrated males and 5 spayed females with an average age of 7.73 years (range: 4.4-10.8) at the time of diagnosis. The DFI > 300 group was also composed of 5 castrated males and 5 spayed females with an average age of 9.96 years (range: 7.1-13.4) at the time of diagnosis. The samples used in the microarray study were a subset of these as described in the "Methods" section. Dog breed, chemotherapy type, tumor phenotype and tumor location are included in Table 1.

### Affymetrix Canine 2.0 Genome Array Analysis

Criteria for assessing differential regulation of probesets were based on the preprocessing algorithm used as both PLIER and RMA have benefits and drawbacks. Briefly, PLIER exhibits higher signal reproducibility and differential sensitivity for low expressing genes yet the variance can be unstable on a log scale, whereas RMA demonstrates fold change compression at the low end of expression, but the variance is stable on a log scale [19]. Thus, selection criteria for genes to validate with qRT-PCR were: PLIER fold change greater than 3 with an uncorrected p-value less than 0.05 and/or RMA fold change greater than 2 with an uncorrected p-value less than 0.05. False discovery rate correction yielded no significant genes so uncorrected p-values were used: this is not surprising in this natural, diverse patient population.

**Figure 1:**
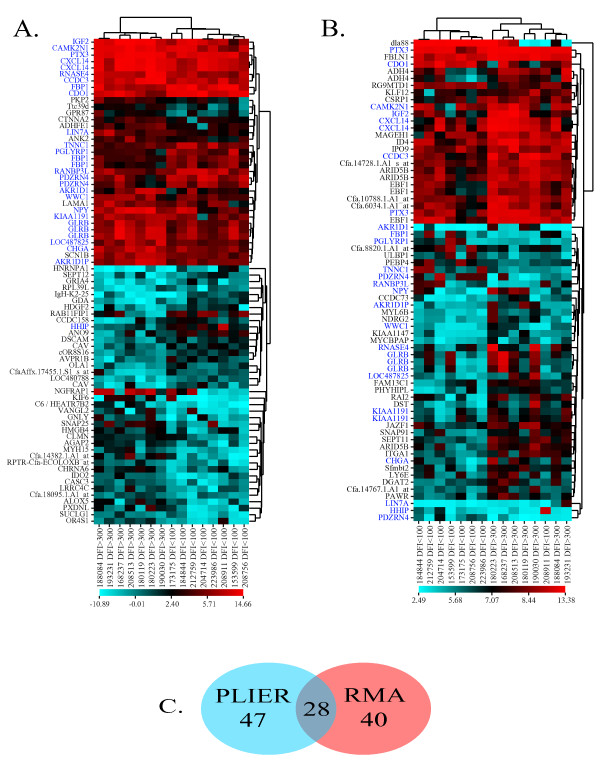
**Fold change analysis of microarray data**. Clustered image maps derived from gene array analysis of 15 canine osteosarcomas. Data was preprocessed with PLIER (A) and RMA (B) algorithms. Probesets were selected based upon fold change > 3 (PLIER) or > 2 (RMA) and an uncorrected p-value < 0.05. (C) Number of probesets meeting selection criteria from each algorithm that were shared and unshared between the two.

Affymetrix Canine 2.0 gene array analysis yielded 75 probesets matching the PLIER criteria and 68 probesets matching the RMA criteria. Twenty-eight probesets and twenty-three genes were shared (Figure 1A &1B, blue labels) between the two selection criteria yielding 115 total probesets for further investigation (Figure 1C). Unsupervised hierarchical clustering of the 75 PLIER-selected probesets grouped the dogs according to their respective disease free interval groups (Figure 1A, X-axis). This hierarchical clustering also grouped the probesets relative to fold change differences between the DFI < 100 day group and the DFI > 300 day group (Figure 1A, Y-axis). This pattern indicates that, based on the genes showing the greatest expression differences, dogs that have a longer disease-free interval (X-axis, left half) have more-similar primary tumor gene expression to each other than to dogs with a short DFI (X-axis, right half), even those of the same breed. Hierarchical clustering of the 68 RMA-selected probesets yielded similar results with all but one of the dogs (208911 DFI < 100) clustering in their respective DFI groups. (Figure 1B). The differences in sample clustering, the ranges of expressed values, and the differences in shared gene clustering (i.e. genes shared between the two algorithms are clustered primarily in half of the PLIER dendogram but spread throughout the RMA dendogram) underscore the fact that different algorithms yield different results and illustrate the value of applying multiple algorithms.

### Quantitative RT-PCR Analysis of Putative Biomarkers and Array Validation

**Figure 2:**
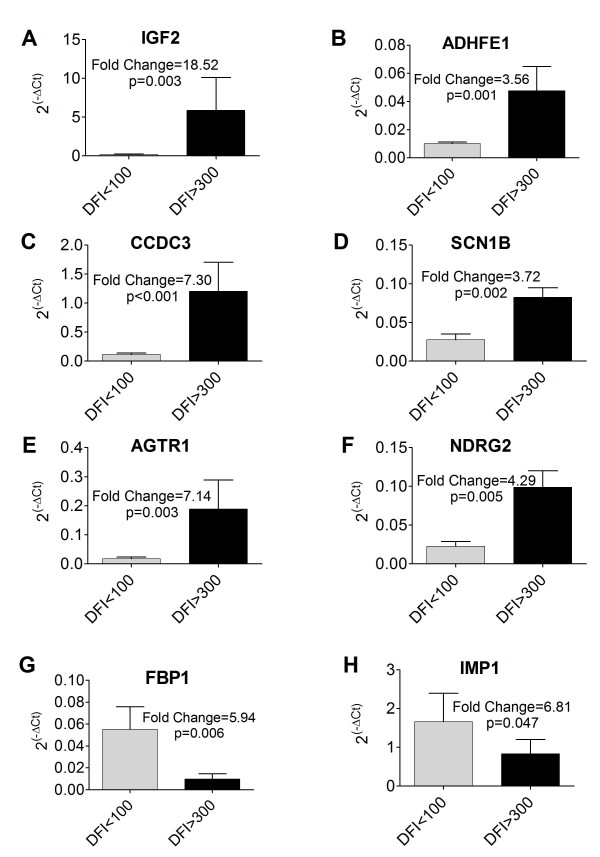
**qRT-PCR validation of genes selected from fold change analysis of microarray data**. Expression represented as 2^(-ΔCt) ^for eight genes selected from fold change analysis of gene array data that were significant on qRT-PCR validation with 20 samples. Higher values indicate higher expression between cohorts and among genes. Fold change and p-values calculated via the REST2009 method. Error bars represent standard error. Insulin-like growth factor II (IGF2, A), alcohol dehydrogenase, iron containing 1 (ADHFE1, B), coiled-coil domain containing 3 (CCDC3, C), sodium channel, voltage-gated, type I, beta (SCN1B, D), angiotensin II receptor, type 1 (AGTR1, E), n-myc downstream-regulated gene family member 2 (NDRG2, F), fructose-1,6-bisphosphatase 1 (FBP1, G), and IGF2 mRNA binding protein 1 (IMP1, H).

Thirty-six genes were assayed for expression via qRT-PCR in 20 OSA samples to both correlate array data to qRT-PCR as well as explore potential biomarker expression via a method not subject to multiple sampling errors. Of these, 8 demonstrated significantly different (p < 0.05) expression between the two cohorts as calculated by both the 2^(-ΔΔCt) ^method [25] with a 2-tailed t-test and the REST2009 [27] iterative method that accounts for amplification efficiency. qRT-PCR expression is plotted as 2^(-ΔCt) ^in Figure 2. Higher expression levels between cohorts and among genes can be visualized as an increased 2^(-ΔCt) ^value. Fold changes and statistical calculations stated in the text were calculated with REST2009 as this program consistently demonstrated higher stringency for significance than the 2^(-ΔΔCt) ^method with t-test.

We observed significant down-regulation of insulin-like growth factor II (IGF2, fold change = 18.52, p = 0.003, Figure 2A) in our poor-responder cohort (DFI < 100). Other significantly down-regulated genes in the DFI < 100 cohort were: alcohol dehydrogenase, iron containing 1 (ADHFE1, fold change = 3.56, p = 0.001, Figure 2B), coiled-coil domain containing 3 (CCDC3, fold change = 7.30, p < 0.001, Figure 2C), sodium channel, voltage-gated, type I, beta (SCN1B, fold change= 3.72, p = 0.002, Figure 2D), angiotensin II receptor, type 1 (AGTR1, fold change = 7.14, p = 0.003, Figure 2E), and n-myc downstream-regulated gene family member 2 (NDRG2, fold change = 4.29, p = 0.005, Figure 2F). Up-regulated genes in the DFI < 100 cohort were: fructose-1,6-bisphosphatase 1 (FBP1, fold change = 5.94, p = 0.006, Figure 2G) and IGF2 mRNA binding protein 1 (IMP1, fold change = 6.81, p = 0.047, Figure 2H). The remaining 28 genes displayed qRT-PCR fold changes similar in amplitude and direction to at least one of the applicable Affymetrix probesets with only one exception. Although these genes did not meet significance criteria on qRT-PCR, there is a strong correlation between the qRT-PCR data and the microarray data (data not shown).

### Pathway Analysis

**Figure 3:**
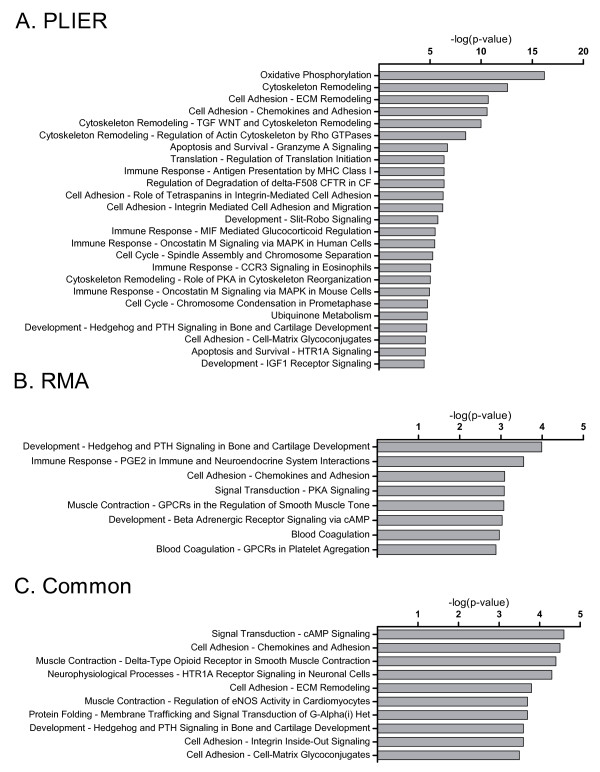
**Pathway analysis, most significant pathways**. Top ranked pathways from GeneGo MetaCore pathway analysis following probeset selection with GEDA's J5 metric. Pathways are ranked based upon p-value, bars represent inverse log of the p-value. (A) Top 25 pathways generated from PLIER preprocessed array data (of 69 meeting significance criteria of p < 0.001). (B) Eight significant pathways generated from RMA preprocessed array data. (C) Top ten significant pathways from an analysis considering only genes common to both RMA and PLIER scoops.

Pathway analysis was utilized to examine the microarray data in a biologically relevant manner and to rule out the false positives commonly found in fold change analysis. To select differentially-expressed genes from the greater-than 40,000 probesets in an unbiased fashion, we utilized the J5 metric as described previously [29]. For the PLIER-processed data, this yielded 3179 total probesets and 1783 unique annotated or identifiable gene identities with human homologs. The RMA-processed data yielded 1374 total probesets with 764 unique identifiers. Probesets that were not associated with a human homolog in the Affymetrix or DAVID databases were hand-annotated, where possible, using NCBI BLAST and/or UCSC BLAT. These datasets were then analyzed with the MetaCore platform to assign functional pathways to each individual dataset as well as to the identifiers common to both PLIER and RMA datasets. Figure 3 displays significantly altered pathways (p < 0.001) by ascending p-value for PLIER (a), RMA (b), and combined RMA/PLIER (c) analyses. Sixty-nine significant pathways were identified using the PLIER dataset (Figure 3A) and eight significant pathways were identified using the RMA dataset (Figure 3B). Analysis of identifiers common to both RMA and PLIER datasets yielded 379 shared identifiers and ten significant pathways (Figure 3C).

The pathway expression differences between good and poor responders primarily involved genes associated with oxidative phosphorylation, bone development, cAMP/Protein Kinase A (PKA) signaling, cell adhesion, cytoskeletal remodeling and immune response. Many of the pathways show modulation in commonly observed "cancer" signatures including matrix metalloproteinases, transforming growth factors, wingless-type MMTV integration site family members (WNTs) and nuclear factor kappa-light-chain-enhancer of activated B cells (NF-κB) downstream targets, as well as actin and myosin cytoskeletal components (data not shown and Additional Files 1, 2).

### qRT-PCR Analysis of the Hedgehog Pathway

**Figure 4:**
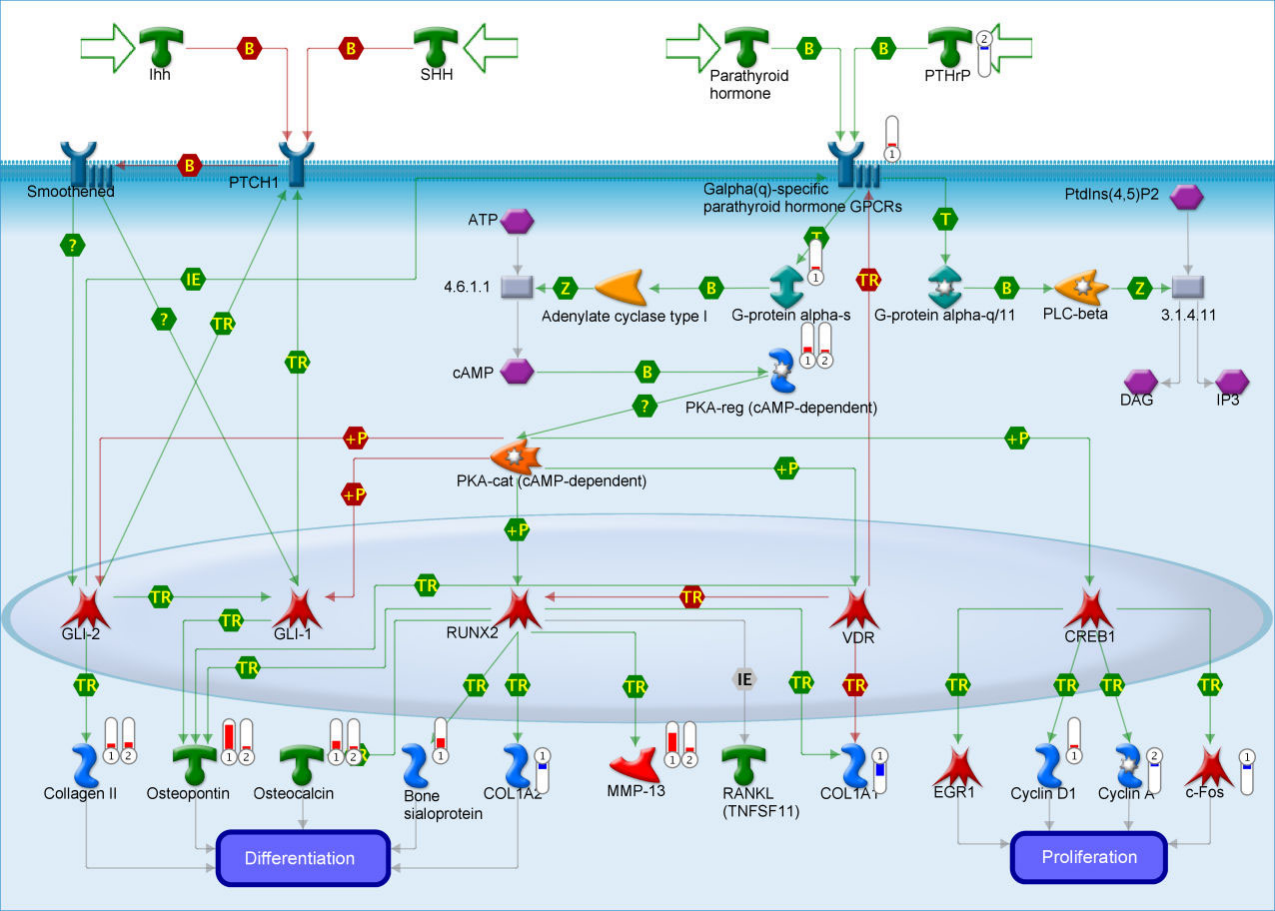
**Hedgehog and Parathyroid Hormone Signaling Pathways in Bone and Cartilage Development**. Red symbols indicate degree of up-regulation of gene target in DFI < 100 days relative to DFI > 300 days, blue symbols indicate relative down-regulation. Numbers in symbols indicate specific array processing algorithm, 1 = PLIER, 2 = RMA.

**Figure 5:**
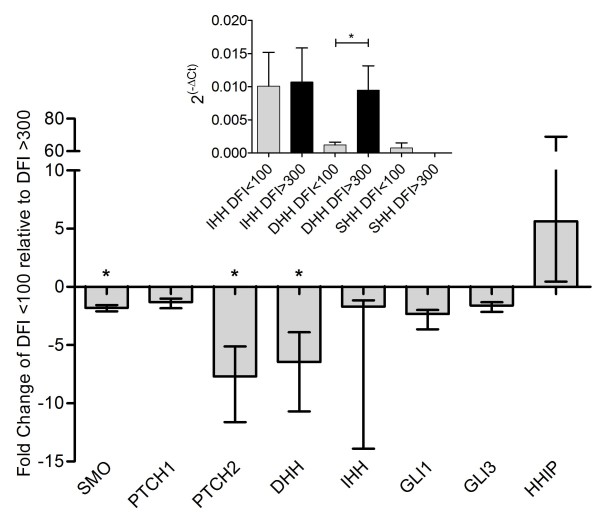
**qRT-PCR analysis of the Hedgehog pathway**. Fold change from qRT-PCR of eight genes in the hedgehog pathway. Genes were selected for analysis based upon significance of the hedgehog pathway in pathway analysis. Fold change calculated via the 2^(-ΔΔCt) ^method (* = p < 0.05). Error bars represent standard deviation and are asymmetrical due to the exponential fold change calculations. Inset: expression represented as 2^(-ΔCt) ^for hedgehog ligands, error bars represent standard error.

The identification of hedgehog pathway components in each pathway (Figure 4) and fold change analysis (HHIP, Figure 1A and 1B), led us to examine expression of nine genes in the pathway via qRT-PCR: hedgehog interacting protein (HHIP), patched (PTCH1 and PTCH2), smoothened (SMO), glioma-associated oncogene family zinc fingers (GLI1 and GLI3), and hedgehog ligands (DHH, SHH, and IHH). Three of these genes, DHH, SMO, and PTCH2, demonstrated significant down-regulation in the poor-responder cohort (Figure 5). Sonic hedgehog was unexpressed in 17 of 20 samples and only minimally expressed in the remaining three (Figure 5 inset).

### Data Classification

Four classification models were generated based on the qRT-PCR gene expression patterns of fifteen genes, the eleven significant genes plus four genes that were members of the Hedgehog signaling pathway or were selected in the fold-change analysis of both the RMA and PLIER normalized data sets: GLI3, HHIP, RAN binding protein 3-like (RANBP3L) and peptidoglycan recognition protein 1 (PGLYRP1). The accuracies for each of these models during model generation and cross validation are listed (Table 3). Stratified cross-validation in each of these models was repeated 10 times using 90% of the samples to train the model and 10% to test the model. The J48 decision tree selected two genes that were most predictive for all samples: ADHFE1 and NDRG2. It successfully predicted cohort for all of the 20 samples. When the same model was built with stratified cross-validation using 90% of samples to train the model and 10% to test the model, it predicted cohort with a 75% success rate. A Support Vector Machine algorithm was used to generate two models. The first, incorporating all 15 genes, predicted cohort with a 100% success rate and 90% in cross-validation. The second SVM model incorporated only the 3 most heavily weighted genes from the previous model, CCDC3, FBP1 and ADHFE1. It also predicted cohort with 100% success rate and 90% in cross-validation. Logistic regression including the three most predictive genes from the SVM model predicted cohort with a 100% success rate and 90% in cross-validation.

**Table 3:** Results of classification modeling.

	Classifier Model			
	J48 Decision Tree (15 Genes)	Support Vector Machine (15 Genes)	Logistic Regression (3 Genes)	Support Vector Machine (3 Genes)
Full Training Set^a^	100%^c^	100%	100%	100%
Stratified Cross-Validation^b^	75%	90%	90%	90%

^a ^Full training sets included data for all 20 samples to both build and test the model.

^b ^Stratified cross-validation models were built with 90% of the samples and tested with the remaining 10% through multiple iterations.

^c ^Percent of samples correctly classified by the model.

## Discussion

In this study, we analyzed gene expression in chemotherapy-naïve primary OSA tumors from 20 dogs with the aim of identifying a gene signature of aggressive metastasis and/or resistance to chemotherapy following definitive treatment with limb amputation and adjuvant therapy with doxorubicin and/or a platinum drug. The purpose of this aim was 3-fold. First, it provides a basis for development of a prognostic screen; such a tool would be of great value to the clinicians diagnosing and treating the more than 8,000 new cases of canine OSA every year. Additionally, pet owners would benefit greatly from a more accurate projected survival time when weighing their dog's quality of life and their own monetary obligations in treatment decisions. Secondly, analysis of gene signatures may allow elucidation of single genes or genetic pathways that may be manipulated for treatment purposes. Finally, dogs are an excellent model for human OSA and identification of markers and pathways leading to disease progression and resistance to therapy in dogs may be translated to the pediatric clinical setting to improve prognosis and treatment of human OSA.

We utilized qRT-PCR to confirm the differential expression of eleven genes between primary OSA from good (DFI > 300 days) and poor (DFI < 100 days) responding dogs (Figs. 2 and 5). Transcriptional profiles of an additional 28 genes selected from fold change analysis of the microarray data were assessed via qRT-PCR and, although differential expression was observed in many, significance criteria for the qRT-PCR analysis were not met (data not shown). Nineteen of these qRT-PCR targets were selected for analysis before pathologist review identified one of the "tumors" as hyperplastic tissue without neoplasia. Removal of that sample and subsequent reprocessing of microarray data removed some of these targets from the RMA and PLIER fold change lists. Despite their failure to achieve significance in our fold-change analysis, the qRT-PCR data for these targets still correlates strongly with the array data on a sample-by-sample basis. Two of these genes, IMP1 and AGTR1, were verified as differentially expressed by qRT-PCR analysis (Figure 2), possibly due to the increased sample number used in each group for this analysis. From the additional 28 genes that failed to show statistically significant differences by qRT-PCR, eleven of these gene targets were selected by the fold change analysis of either RMA or PLIER processed data shown in Figure 1. The failure of these gene targets to reach significance in the qRT-PCR analysis may reflect the variability in microarray preprocessing algorithms as well as differences in expression values based on primer design as primers used in this study were not designed to align with Affymetrix probe locations. In addition, since our qRT-PCR analysis used a larger number of samples than the microarray study, some of the microarray hits may have been false positives that have now been removed from the list of putative biomarkers thanks to the qRT-PCR analysis.

Although these genes were primarily assessed by qRT-PCR for their prognostic potential, they may also have functional roles in metastatic progression and resistance to chemotherapy. IMP1 (6.93 fold up-regulated in the poor-responders), also known as IGF2BP1 and not to be confused with the family of IGF binding proteins, is a member of a family of three oncofetal proteins (IMP1-3) whose function is to bind and regulate mRNA stability in the cytoplasm during development. IMP1 expression is stimulated by Wnt/β-catenin signaling and has many regulatory targets, some of which are implicated in cancer: stabilization of c-myc [34,35] and CD44 mRNAs [36], translational suppression of IGF2 [37], and localization of β-actin mRNA to sites of actin polymerization [38]. These targets can affect cell growth and survival as well as metastatic mechanisms such as invadopodia formation and cell adhesion [39]. IMP1 over-expression has been associated with poor prognosis in numerous cancer types including human ovarian and colorectal carcinomas [39,40].

IGF2 (15.4 fold down-regulated in the poor-responders) has been shown to be down-regulated in response to IMP1 as well as to hedgehog pathway inhibition and the observed alterations in these factors/pathways may account for some down-regulation of IGF2 [41]. Additionally, IGF2 expression is modulated by numerous other factors including parathyroid hormone (PTH), cortisol, and transforming growth factor beta (TGF-β) [42]. Finally, our pathway analysis shows reduction in PTH related protein (PTHrP) and subsequent modulation of the PTH pathway suggesting IGF2 may be comparatively under-expressed in poor-responders due to decreased PTHrP expression in that cohort. It is important to note that the observed down-regulation of IGF2 (and all other genes discussed here) is relative between cohorts and that the mRNA was expressed in all samples, but to a lesser degree in poor-responders.

FBP1 (5.94 fold up-regulated in the poor-responders) is involved in gluconeogenesis and is expressed in the liver and, to a lesser extent, most other cell types. Its action opposes that of phosphofructokinase and its expression can lead to increased cellular glutathione and an apoptosis-resistant phenotype [43]. Bigl and colleagues examined FBP1 expression in several types of breast cancer and found it to be up-regulated in invasive lobular carcinoma when compared to normal tissue but down-regulated in other tumor types suggesting a variable role depending on tumor type [44].

ADHFE1 is an iron-activated alcohol dehydrogenase with widely conserved motifs that is found in multiple tissue types. It has been shown to oxidize gamma-hydroxybutyrate and is 3.50 fold down-regulated in the poor-responders [45]. CCDC3, also down-regulated in the poor-responders (7.10 fold), encodes a 270 amino acid protein with a putative coiled-coil domain near the C-terminus. Recent reports indicate that this protein is secreted by both adipocytes and endothelial cells and is under both hormonal and nutritional control [46]. Interestingly, CCDC3 was identified as a factor contributing to ifosfamide resistance in a mouse xenograft model using human OSA cell lines. Bruheim and colleagues reported a 40-fold down-regulation of this gene in resistant tumor cells[47]. As none of the dogs in the current study received ifosfamide, this gene may contribute generally to both metastasis and chemotherapeutic resistance.

Chioni *et al*. recently elucidated a role for SCN1B in cellular adhesion and migration in breast cancer cell lines. Their mildly metastatic cell line demonstrated increased expression of SCN1B compared to the highly metastatic cell line; furthermore, siRNA-mediated knockdown of SCN1B decreased adhesion and increased migration in the mildly metastatic line [48]. Our observed 3.70 fold down-regulation of SCN1B in the poor-responders indicates that the tumor environment may become more pro-migratory due to reduced expression of this factor.

The putative tumor suppressor gene NDRG2 (4.57 fold down-regulated in the poor-responders) is expressed in an inverse relationship to proliferation in normal tissues and has been observed to be down-regulated in numerous tumor types, especially in response to myc oncogene expression (See [49] for review). Recent cytogenetic analysis of canine OSA revealed breed independent myc amplification in 40% of the cases, suggesting this is a common chromosomal aberration in both canine [9] and human OSA [50]. Tepel and colleagues demonstrated epigenetic promoter modifications as a mechanism for suppression of this gene in glioblastoma [51]. Recent evidence has identified numerous mechanisms by which NDRG2 acts as a tumor suppressor and invasion attenuator: anti-proliferative suppression of AP-1 in colorectal carcinoma [52], anti-invasive suppression of NF-κB in fibrosarcoma and melanoma cell lines [53], pro-apoptotic involvement in the p53 pathway [54], and reduction in invasion and intracellular β-catenin in NDRG2-transfected cell lines [55]. Kim and colleagues demonstrated that NDRG2 expression decreases with increasing tumor stage in colon carcinoma, indicating that this may be an excellent marker for molecular staging [55]. Furthermore, recent studies have shown that the myc oncogene stimulates mitochondrial glutaminolysis resulting in reprogramming of mitochondrial metabolism to depend on glutamine catabolism to sustain cellular viability [56]. In support of this hypothesis, our pathway analysis associated both upregulation of the myc oncogene and alterations in mitochondrial oxidative phosphorylation with poor outcome.

To identify the prognostic potential of these genes, we built several classification models to identify genes with the most predictive power. Of the four models tested, all classified the samples with 100% accuracy when the model was built from all 20 samples. However, when stratified cross-validation was used, the two SVMs and the linear regression model were 90% correct whereas the J48 decision tree was only 75% correct. These stratified cross-validation results are generally thought to more accurately reflect results in subsequent applications of the model. The two SVM models classified with the same success rate regardless of whether built with all fifteen genes or the three most heavily weighted contributors, suggesting that CCDC3, ADHFE1 and FBP1 are highly predictive in this data set and are likely to be robust classifiers in future OSA studies.

While biomarker identification can be successful using traditional fold change methodology, as evidenced by our gene hits above, understanding of the processes of metastasis and chemoresistance can be furthered by all-inclusive pathway analysis. Thus, to eliminate some of the arbitrary nature of traditional fold change analysis, we also examined our microarray data via this methodology. Over 4,000 probesets were selected from microarray data using the J5 metric, annotated and converted to human identifiers using public-access tools including DAVID, and assigned to pathways with the GeneGo MetaCore program. This program assigns pathway significance based upon the number of genes represented within a pathway and the direction of change. The overwhelming benefit to this methodology is that change in a single gene will be ignored unless related genes also demonstrate altered expression. Thus, the downstream impact of chip anomalies, probeset inefficiencies and differences in preprocessing algorithms can be dramatically reduced. This type of analysis allows integration of the typical microarray methodology examining highly expressed genes with the systems biology approach of examining large numbers of genes, some of which may be expressed only at low levels despite their importance to a given pathway [28]. One pertinent example is PTEN deletion which was identified as a chromosomal aberration in 40% of canine OSA [9]. However, loss of PTEN was not detected in our fold change analysis, but was associated with poor outcome by pathway analysis (Additional File 2).

Although discussing each of the modulated pathways is beyond the scope of this study, some notable generalizations can be addressed. Cell adhesion and cytoskeletal remodeling are both strongly represented in pathways we have identified as significantly altered between our two cohorts; this suggests that the aggressiveness of tumor cells with regard to these two elements of metastasis may be just as important as chemoresistance mechanisms in this population. Bone-related developmental and immune-response pathways are also represented, much as one would expect in these osteoblastic/osteolytic tumors. Finally, cAMP/PKA signaling pathways also have strong representation in these analyses. Similar alterations in cAMP/PKA signaling with upregulation of the PKA regulatory subunit 1α have been described in other cancers [57]. However, the differences between good and poor responders are notable and provide evidence for variation in molecular phenotype contributing to aggressiveness within the same histologic subtype of tumor.

The pathway analysis converged with the traditional fold change analysis at the hedgehog signaling pathway. The hedgehog signaling pathway appears to act upstream of Wnt/β-catenin signaling during bone development and aberrant hedgehog signaling has been associated with cancer development and progression [58]. As a result, we decided to examine other genes in the hedgehog pathway with qRT-PCR. Of the eight hedgehog-related genes examined, three were significantly down regulated in the poor responder cohort. These three genes, SMO, PTCH2 and DHH, where not identified on traditional fold change analysis and this is likely due to two factors. First, DHH is not annotated in the canine genome so primers were designed based on a region of the canine genome homologous to the gene in other species. Considering this, the Canine 2.0 microarray does not have a probe for this gene. Probes were present for the SMO and PTCH2 genes, however, in this study, raw array expression values for these genes were very low suggesting that the signal may be nearing the detection limit of the microarrays.

Hedgehog interacting protein (HHIP) was identified by fold change criteria in both RMA and PLIER preprocessed arrays. The up-regulation in the poor responder cohort was also observed as a trend in qRT-PCR but did not meet significance criteria. HHIP antagonizes all three of the hedgehog family of ligands (SHH, DHH and IHH) and has been shown to be down-regulated in numerous epithelial tumor types [59] with the notable exception of basal cell carcinoma where it is upregulated [60]. HHIP is also abundant in endothelial cells but is suppressed during angiogenesis through a VEGF mediated pathway [61]. The up-regulation of HHIP observed in our poor-responders likely has some causative relationship to the down-regulation of DHH and, through feedback loops, SMO and PTCH2 in the same cohort.

Three studies have examined gene expression in primary human OSA to identify chemotherapy-resistance signatures by comparing good and poor responders [62-64]. Among them, they identified over 200 differentially regulated genes but each gene set was unique to each study (i.e. there was no overlap in expression signatures). More recently, Walters and colleagues [65] assayed expression patterns in OSA cell lines with differing aggressiveness and identified 252 differentially regulated genes, four of which overlapped with the Mintz *et al*. study's gene signature [63]. This lack of similarity in expression patterns is observed in array analyses of various tumor types and is not at all surprising when one contemplates the differences in array preprocessing algorithms. Considering the disparity between the heat maps presented in Figure 1A and 1B, it is plausible that the exact same data processed in two different ways may yield two very different sets of candidate genes. Thus, in addition to traditional fold change analysis of microarray data for biomarker identification, a broader, unbiased systems-biology approach, such as we have done here, is likely to identify biological changes that can be reliably verified in multiple data sets. In fact, this approach was used to analyze multiple independent data sets to show that genes involved in the oxidative phosphorylation pathway were reduced in metastases compared to primary solid carcinomas [28]. Interestingly, in the current comparison of primary sarcomas, increased expression of genes in the oxidative phosphorylation pathway was associated with a poor outcome, suggesting that different metabolic factors may contribute to the initiation of metastasis from a primary tumor and the implantation and successful growth of metastasis at a distant site.

Given the small sample size of the study, we acknowledge that this data serves primarily as a road map for future studies. Our sample size was small primarily due to the stringent selection criteria set forth in Methods limiting our samples to dogs that had appendicular osteosarcoma, undergone amputation and received chemotherapy. Furthermore, we limited our samples to those from dogs with either very low or very high DFIs: the 100 and 300-day cutoffs were intended to straddle our facility's average DFI of 200 days. Selvarajah and colleagues recently studied gene expression in a group of dogs with OSA with good and poor outcome [66]. They utilized a larger sample size (n = 32) but included dogs with axial OSA as well as dogs that did not receive adjuvant chemotherapy. Although they did not observe differences in outcome due to these factors, previous studies have established an effect on DFI [2,3,5,7]. They also based their good and poor responder groups on survival time instead of DFI: this can greatly affect outcome groups in a field of medicine where euthanasia is practiced. Beyond study-design differences, we applied a systems-based model for biomarker/pathway discovery by using the J5 metric to enrich for high to medium expressing genes that are most appropriate for selection as diagnostic/prognostic biomarkers, as opposed to fold-change based input. Hand annotation of many probesets based upon sequence homology allowed us to input a very large and complete data set into the MetaCore pathway analysis. Despite these differences in study design and analysis methods, we identified some pathways with similarity to those they identified in their PANTHER analysis, including hedgehog signaling, WNT signaling and chemokine signaling. Considering the differences in chemotherapy requirements between the two studies, these pathways may be most indicative of increased metastatic potential as opposed to chemotherapeutic resistance.

Work by Paoloni and colleagues provides strong evidence for the validity of dogs as a model for human OSA. They found that canine and human OSA are more similar to each other than to normal tissues from the same species [10]. This, in concert with our growing body of knowledge regarding gene and pathway derangements in canine OSA provides insights into the mechanisms of OSA progression and chemoresistance.

## Conclusions

The present study has examined gene expression in primary canine OSA via both traditional fold change analysis and systems-based pathway analysis and found significant differences between dogs that responded poorly to chemotherapy following definitive treatment and dogs that responded well as evidenced by a long disease-free interval. This study has identified candidate biomarkers of aggressive tumors as well as pathways that are deranged in poor responders relative to good responders, opening the door for molecular prognostic screening in canine OSA and further molecular comparison between the human and canine disease. Although further studies, such as protein expression analysis will be necessary to solidify the role of these genes and pathways in OSA, targets identified here provide a strong foundation from which to identify druggable targets and markers of progression in OSA.

## Competing interests

The authors declare that they have no competing interests.

## Authors' contributions

LEO carried out all sample preparation and molecular genetics studies (excepting Affymetrix methods), analyzed data, performed pathway and statistical analyses and drafted the manuscript. AAP performed preliminary pathway analysis and taught LEO the methodology. DAK performed pathology review and reviewed hospital records for histology interpretation. JS created classification models. RST performed preliminary microarray data analysis and taught LEO the methodology. DLD conceived of the study design, participated in its coordination and helped to draft the manuscript. All authors read and contributed to the final manuscript.

## Pre-publication history

The pre-publication history for this paper can be accessed here:

http://www.biomedcentral.com/1471-2407/10/506/prepub

## Supplementary Material

Additional file 1 **Signal transduction - cAMP signaling**. Top scored pathway map in the analysis of gene targets common to both PLIER and RMA processing. Red symbols indicate degree of upregulation of gene target in DFI < 100 days relative to DFI > 300 days, blue symbols indicate relative down-regulation. Numbers in symbols indicate specific array processing algorithm, 1 = PLIER, 2 = RMA. Click here for file

Additional file 2 **Cell Adhesion- Chemokines and Adhesion.** Second scored pathway for the analysis of gene targets common to both PLIER and RMA processing. Red symbols indicate degree of upregulation of gene target in DFI < 100 days relative to DFI > 300 days, blue symbols indicate relative down-regulation. Numbers in symbols indicate specific array processing algorithm, 1 = PLIER, 2 = RMA. Click here for file
