# Supplementary figures and images for: Expression profiling in canine osteosarcoma: identification of biomarkers and pathways associated with outcome
Source: BMC Cancer. 2010 Sep 22;10:506. doi: 10.1186/1471-2407-10-506 (PMC2955038; doi:10.1186/1471-2407-10-506)

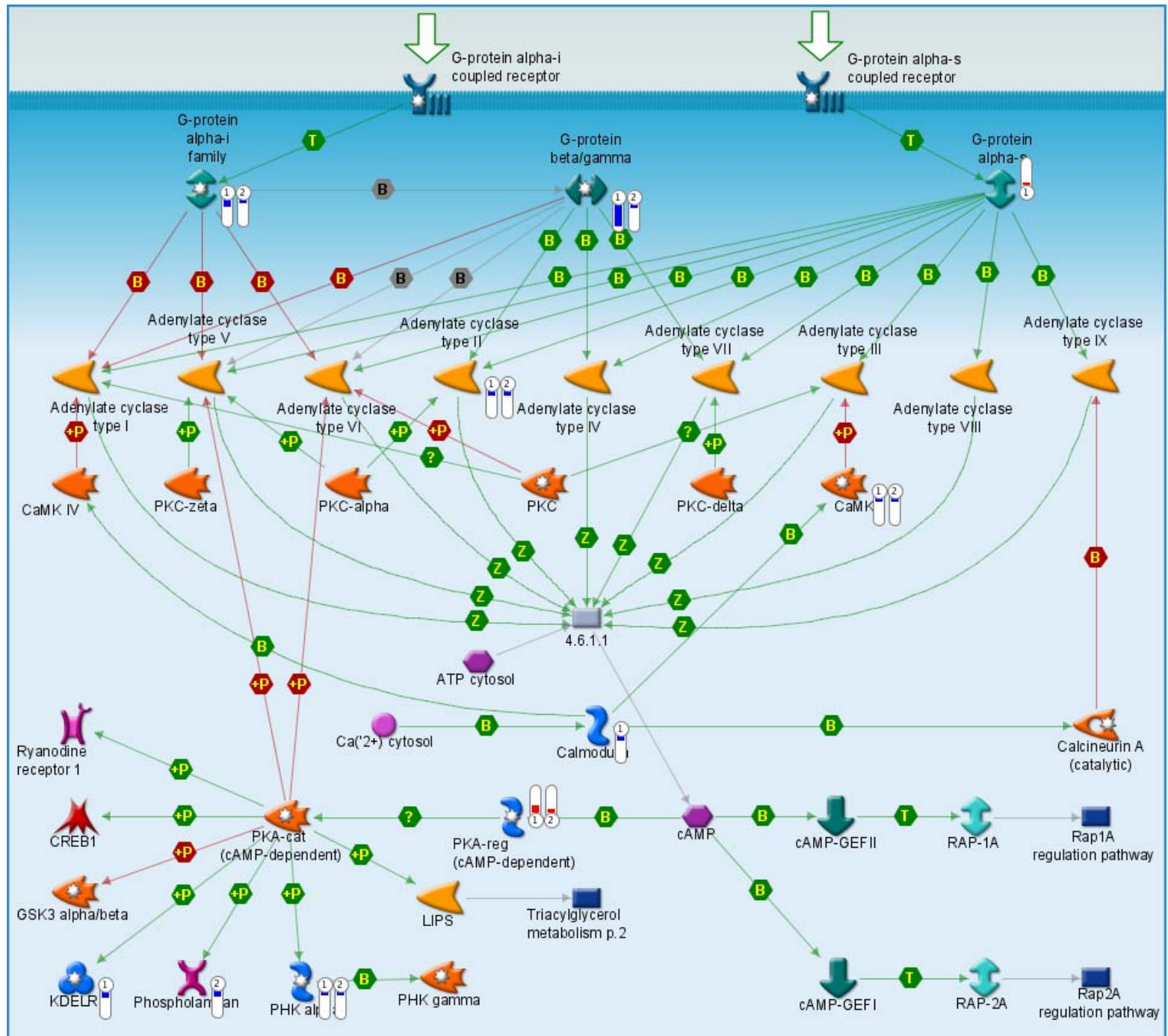

Supplement: Additional file 1 — Signal transduction - cAMP signaling. Top scored pathway map in the analysis of gene targets common to both PLIER and RMA processing. Red symbols indicate degree of upregulation of gene target in DFI < 100 days relative to DFI > 300 days, blue symbols indicate relative down-regulation. Numbers in symbols indicate specific array processing algorithm, 1 = PLIER, 2 = RMA. [file 1471-2407-10-506-S1.PDF]

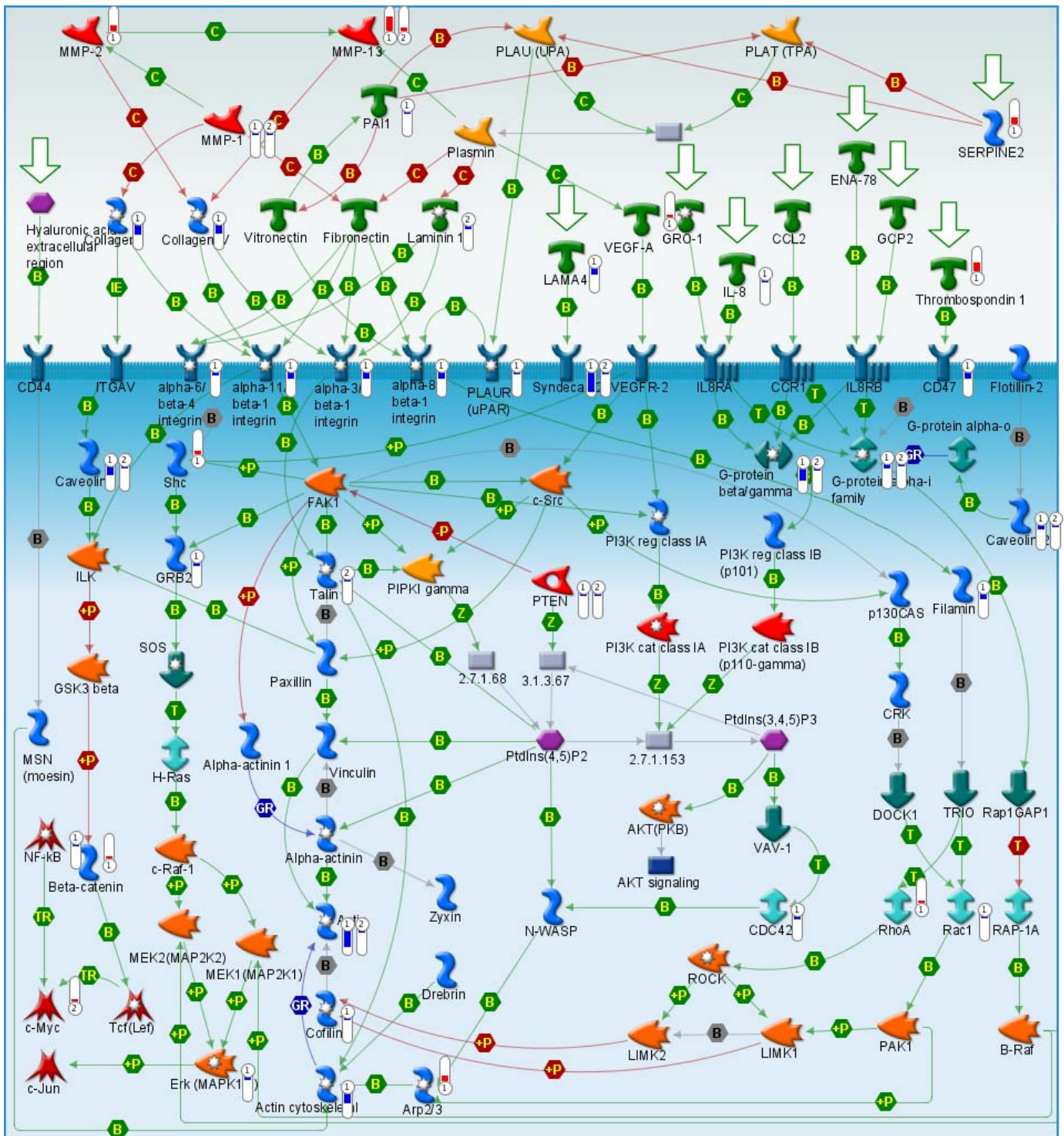

Supplement: Additional file 2 — Cell Adhesion- Chemokines and Adhesion. Second scored pathway for the analysis of gene targets common to both PLIER and RMA processing. Red symbols indicate degree of upregulation of gene target in DFI < 100 days relative to DFI > 300 days, blue symbols indicate relative down-regulation. Numbers in symbols indicate specific array processing algorithm, 1 = PLIER, 2 = RMA. [file 1471-2407-10-506-S2.PDF]
